# Supplementary material for: Do Determinants of Quality of Life Differ in Older People Living in the Community and Nursing Homes?
Source: Int J Environ Res Public Health. 2023 Jan 4;20(2):916. doi: 10.3390/ijerph20020916 (PMC9858919; doi:10.3390/ijerph20020916)
Supplement: Supplementary file 1 [file ijerph-20-00916-s001.zip › ijerph-2087803-supplementary.pdf]

Supplementary Table S1. Relationships of EuroQol-5D test dimensions to age, nutritional parameters and PA in NH residents.

	Nursing Homes Residents														
	Mobility			Self-Care			Usual Activities			Pain/Discomfort			Anxiety/Depression		
	No Problems	Problems	*p* Value	No Problems	Problems	*p* Value	No Problems	Problems	*p* Value	No Problems	Problems	*p* Value	No Problems	Problems	*p* Value
Age	71.5 (64.0;80.0)	76.5 (68.0;84.0)	0.048	72.0 (66.0;82.0)	74.0 (68.0;84.0)	ns	72.0 (64.0;82.0)	75.0 (68.0;82.0)	ns	68.0 (64.0;74.0)	75.0 (67.0;83.0)	ns	68.0 (64.0;80.0)	75.0 (68.0;83.0)	0.034
MNA	22.5 (20.5;24.5)	19.8 (17.5;23.5)	0.014	22.5 (19.0;24.0)	21.0 (18.5;23.0)	ns	21.5 (19.0;23.5)	21.0 (18.5;23;5)	ns	23.0 (21.0;25.5)	20.5 (18.5;23.5)	0.004	23.0 (20.5;24.5)	20.5 18.0;23.0)	0.004
BMI	25.9 (22.3;30.8)	27.7 (23.0;32.7)	ns	27.1 (23.1;30.8)	25.8 (21.8;33.6)	ns	26.1 (23.1;30.4)	27.5 (22.0;32.9)	ns	26.8 (23.9;31.0)	26.6 (22.3;31.3)	ns	25.0 (21.4;30.4)	27.5 (23.8;32.0)	ns
WHtR	0.60 (0.54;0.66)	0.60 (0.54;0.69)	ns	0.60 (0.55;0.65)	0.60 (0.53;0.70)	ns	0.60 (0.55;0.65)	0.60 (0.53;0.69)	ns	0.60 (0.54;0.67)	0.60 (0.54;0.66)	ns	0.57 (0.52;0.62)	0.61 (0.55;0.65)	ns
Waist (cm)	94.0 (84.0;101)	92.0 (84.0;105)	ns	94.0 (86.0;102)	93.0 (82.0;105)	ns	94.0 (84.0;102)	93.0 (84.0;103)	ns	98.0 (87.0;107)	92.0 (84.0;102)	ns	94.0 (82.0;103)	93.0 (84.0;103)	ns
Calf (cm)	34.7 (32.0;38.0)	34.8 (32.0;37.0)	ns	34.5 (32.0;38.0)	36.0 (32.0;37.0)	ns	34.5 (33.0;37.0)	36.0 (31.0;37.0)	ns	35.0 (32.0;37.0)	34.5 (32.0;37.0)	ns	34.5 (33.0;37.0)	35.0 (33.0;37.0)	ns
FFM (%)	62.2 (56.7;72.0)	61.2 (51.6;65.8)	ns	62.1 (55.4;69.9)	61.1 (53.1;65.7)	ns	62.0 (56.7;69.9)	61.1 (53.1;68.8)	ns	60.9 (55.4;68.9)	62.0 (55.4;68.8)	ns	66.4 (59.8;74.1)	60.3 (54.4;65.1)	0.005
FM (%)	37.9 (28.0;43.8)	38.7 (34.2;46.9)	ns	38.0 (30.1;44.6)	38.7 (34.4;43.3)	ns	38.0 (30.1;43.0)	39.0 (32.0;47.7)	ns	40.2 (33.1;49.1)	37.0 (30.1;44.4)	ns	34.7 (28.0;42.4)	39.3 (34.8;45.1)	0.005
Body volume (Lt)	62.4 (54.1;77.8)	67.4 (55.3;77.4)	ns	66.8 (56.6;77.1)	62.1 (54.1;81.0)	ns	64.2 (55.0;77.8)	67.9 (55.3;77.4)	ns	72.6 (56.3;78.6)	64.2 (54.1;77.4)	ns	66.0 (55.0;78.6)	64.5 (55.4;76.1)	ns
Body density	1.01 (1.00;1.04)	1.01 (1.00;1.02)	ns	1.01 (1.00;1.03)	1.01 (1.00;1.03)	ns	1.01 (1.00;1.03)	1.01 (1.00;1.03)	ns	1.01 (1.00;1.03)	1.01 (1.00;1.03)	ns	1.02 (1.00;1.04)	1.01 (1.00;1.02)	0.005
RMR (kcal/kg)	19.2 (16.3;22.5)	18.5 (16.0;20.7)	ns	18.8 (16.3;21.4)	18.9 (15.5;21.3)	ns	19.0 (16.8;21.0)	18.7 (15.8;21.5)	ns	18.9 (16.8;21.3)	18.8 (16.1;21.4)	ns	19.8 (17.9;24.1)	18.7 (16..1;20.5)	ns
TBW (%)	51.0 (46.1;55.2)	48.8 (45.0;52.7)	ns	50.5 (45.9;53.5)	49.6 (45.1;53.1)	ns	50.8 (45.9;53.1)	49.2 (45.0;55.3)	ns	47.7 (45.4;52.5)	50.0 (46.1;53.3)	ns	52.0 (47.2;56.1)	48.8 (45.1;52.7)	ns
ECW/ICW	0.90 (0.84;0.94)	0.91 (0.85;0.95)	ns	0.91 (0.85;0.94)	0.91 (0.84;0.96)	ns	0.92 (0.87;0.95)	0.89 (0.83;0.94)	ns	0.89 (0.84;0.93)	0.91 (0.85;0.95)	ns	0.90 (0.80;0.94)	0.91 (0.87;0.95)	ns
BCM (%)	34.3 (31.8;38.1)	33.7 (30.2;36.6)	ns	34.3 (31.3;37.7)	33.8 (29.8;36.3)	ns	34.3 (31.8;37.1)	33.8 (30.9;37.7)	ns	33.2 (30.8;36.5)	34.4 (31.7;37.7)	ns	36.3 (33.6;40.3)	33.4 (31.8;36.5)	0.047
SMI (kg/m^2)	7.6 (6.8;8.5)	8.3 (7.5;9.3)	ns	7.6 (6.7;9.2)	7.6 (6.3;8.6)	ns	7.4 (6.4;8.6)	7.9 (6.9;8.8)	ns	7.9 (6.2;8.5)	7.5 (6.9;8.9)	ns	8.0 (6.9;9.4)	7.5 (6.7;8.6)	ns
Z (Ω)	540 (493;611)	550 (469;626)	ns	532 (485;611)	553 (506;620)	ns	556 (487;620)	545 (475;598)	ns	582 (485;643)	545 (486;598)	ns	532 (478;618)	553 (490;613)	ns
R (Ω)	537 (489;607)	546 (466;623)	ns	526 (482;607)	550 (500;617)	ns	553 (483;617)	539 (473;595)	ns	578 (483;639)	539 (483;595)	ns	526 (474;610)	550 (487;609)	ns
Xc (Ω)	68.1 (60.1;73.6)	60.7 (49.8;78.3)	ns	66.2 (55.8;73.4)	67.7 (57.2;79.9)	ns	67.3 (57.0;73.1)	64.6 (55.8;78.3)	ns	67.3 (55.5;78.2)	66.3 (56.4;73.7)	ns	67.3 (60.1;73.7)	66.3 (56.2;74.2)	ns
PhA	7.14	6.61	0.044	6.89	6.65	ns	6.86	6.84	ns	6.72	6.86	ns	7.24	6.75	ns

	(6.38;7.71)	(5.91;7.37)		(6.19;7.53)	(6.08;7.86)		(6.15;7.51)	(6.11;7.86)		(5.72;7.53)	(6.19;7.58)		(6.13;7.94)	(6.15;7.38)	
PA-EE	34.4 (32.6;37.8)	33.9 (32.6;36.9)	ns	34.5 (32.8;38.3)	33.4 (31.9;36.0)	ns	34.4 (32.6;38.4)	33.7 (32.4;36.8)	ns	34.0 (33.1;35.5)	34.2 (32.4;37.4)	ns	35.5 (33.9;39.4)	33.7 (31.9;36.0)	0.0004
PA-HRB	1.00 (0.00;2.00)	0.50 (0.00;1.00)	0.004	1.0 (0.0;2.0)	1.0 (0.0;1.0)	ns	1.0 (0.0;2.0)	1.0 (0.0;2.0)	ns	2.0 (0.0;2.0)	1.0 (0.0;2.0)	ns	2.0 (0.0;2.0)	1.0 (0.0;1.0)	0.005

MNA: Mini Nutritional Assessment; BMI: body mass index; WHtR: waist-to-height ratio; FM: fat mass; FFM: fat-free mass; RMR: resting metabolic rate; TBW: total body water; ECW/ICW: extracellular water and intracellular water ratio; BCM: body cell mass; SMI: skeletal muscle index; Z: impedance; R: resistance; Xc: reactance; PhA: phase angle; Ω: Ohm; VAS scale: visual analogue scale; PA-EE: physical activity energy expenditure; PA-HRB: physical activity health related behaviours; NH: nursing home group; CD: community- dwelling group; ns: not significant.

Supplementary Table S2. Relationships of EuroQol-5D test dimensions to age, nutritional parameters and PA in CD older subjects.

.	**Community Dwelling**														
	**Mobility**			**Self-Care**			**Usual Activities**			**Pain/Discomfort**			**Anxiety/Depression**		
	**No Problems**	**Problems**	***p*-Value**	**No Problems**	**Problems**	***p*-Value**	**No Problems**	**Problems**	***p*-Value**	**No Problems**	**Problems**	***p*-Value**	**No Problems**	**Problems**	***p*-Value**
Age	74.0 (66.0;83.5)	75 (70.5;82.0)	ns	74.0 (66.0;82.5)	78.5 (68.0;84.5)	Ns	74.0 (66.5;82.5)	76.0 (66.5;83.5)	ns	75.0 (71.0;82.0)	75.0 (66.0;83.0)	ns	74.0 (67.0;79.0)	75.0 (66.0;84.0)	ns
MNA	27.0 (25.5;28.3)	24.8 (22.8;27.0)	0.002	26.50 (24.3;28.0)	24.0 (21.3;25.5)	0.001	27.0 (24.8;28.5)	25.3 (22.8;27.0)	0.005	28..0 (26.0;29.0)	25.5 (23.5;27.5)	0.012	28.0 (25.5;28.5)	25.5 (23.5;27.5)	0.021
BMI	27.2 (25.2;29.2)	29.7 (25.8;33.1)	0.015	27.5 (25.2;30.5)	29.7 (25.7;32.1)	ns	27.5 (25.8;30.5)	29.5 (24.7;31.2)	ns	27.3 (25.5;29.0)	28.7 (25.2;31.0)	ns	27.3 (24.4;28.1)	29.0 (25.7;31.6)	0.022
WHtR	0.58 (0.54;0.62)	0.61 (0.55;0.65)	0.04	0.58 (0.54;0.64)	0.61 (0.57;0.66)	ns	0.58 (0.55;0.64)	0.59 (0.54;0.64)	ns	0.57 (0.56;0.61)	0.59 (0.54;0.64)	ns	0.56 (0.53;0.58)	0.61 (0.55;0.64)	0.014
Waist (cm)	94.0 (86.5;100)	93.5 (87.5;102.5)	ns	93.5 (86.5;101)	95.0 (88.0;102)	ns	95.0 (87.5;102)	93.0 (86.5;99.0)	ns	94.0 (84.8;100)	94.0 (87.5;102)	ns	88.5 (84.0;100)	95.0 (88.0;102)	ns
Calf (cm)	36.5 (35.0;38.3)	37.0 (35.0;40.0)	ns	36.8 (35.0;39.0)	37.0 (34.3;39.0)	ns	36.5 (35.0;39.0)	37.0 (35.0;39.0)	ns	36.3 (34.5;38.0)	37.0 (35.0;39.0)	ns	36.0 (35.0;38.0)	37.0 (35.0;39.0)	ns
FFM (%)	66.4 (60.1;72.1)	59.8 (54.4;64.3)	<0.0001	62.4 (59.1;68.6)	59..4 (54.9;65.2)	ns	62.2 (58.6;68.5)	61.7 (55.7;68.4)	ns	66.3 (61.2;72.0)	60.9 (56.0;68.3)	0.015	67.6 (61.6;72.9)	60.4 (55.8;66.6)	0.0007
FM (%)	34.8 (28.3;40.4)	39.9 (35.2;46.3)	<0.0001	37.8 (31.6;41.1)	39.9 (34.8;46.0)	ns	39.1 (32.0;41.1)	36.5 (31.1;44.3)	ns	34.8 (28.9;40.3)	39.0 (31.7;44.2)	0.015	34.8 (28.9;40.3)	39.0 (28.9;40.3)	0.0007
Body volume (Lt)	70.7 (61.8;78.4)	68.9 (62.0;79.0)	ns	70.3 (62.0;87.8)	67.9 (61.0;76.0)	ns	71.3 (63.9;81.9)	68.2 (59.7;75.9)	ns	69.4 (61.3;78.9)	69.6 (62.0;78.4)	ns	67.6 (59.2;79.1)	70.1 (62.2;77.9)	ns
Body density	1.02 (1.01;1.04)	1.01 (1.00;1.02)	<0.0001	1.02 (1.01;1.03)	1.01 (1.00;1.02)	ns	1.01 (1.01;1.03)	1.01 (1.00;1.03)	ns	1.02 (1.01;1.04)	1.01 (1.00;1.03)	0.019	1.03 (1.01;1.04)	1.01 (1.00;1.02)	0.001
RMR (kcal/kg)	18.5 (17.7;20.3)	17.3 (15.6;19.4)	0.001	18.2 (17.2;20.1)	17.2 (15.7;19.8)	ns	18.0 (17.1;19.8)	18.1 (16.2;20.3)	ns	18.7 (18.0;19.8)	17.9 (16.4;20.1)	ns	19.4 (18.2;20.2)	17.7 (16.1;20.0)	0.002
TBW (%)	53.4 (49.8;57.4)	48.7 (47.4;52.2)	0.0002	51.7 (47.8;55.2)	49.0 (47.6;53.4)	ns	51.4 (47.7;54.5)	50.1 (47.8;54.7)	ns	53.2 (51.1;58.1)	50.3 (47.5;54.1)	0.017	53.7 (51.7;56.8)	50.1 (47.4;53.9)	0.0015
ECW/ICW	0.90 (0.84;0.91)	0.90 (0.86;0.92)	0.012	0.88 (0.80;0;92)	0.90 (0.86;0.93)	ns	0.88 (0.78;0.91)	0.90 (0.85;0.92)	0.034	0.79 (0.75;0.92)	0.89 (0.84;0.92)	ns	0.86 (0.75;0.92)	0.89 (0.84;0.92)	ns
BCM (%)	36.9	33.4	0.0001	34.8	33.6	ns	34.8	34.4	ns	36.4	34.3	0.038	37.5	34.0	0.001

	(33.8;40.1)	(31,8;35.5)		(32.8;38.3)	(32.2;36.7)		(33.0;38.2)	(32.2;38.0)		(33.6;41.1)	(32.2;37.5)		(34.7;40.7)	(32.1;37.7)	
SMI (kg/m²)	8.8 (7.6;10.8)	8.3 (7.5;9.3)	0.017	8.5 (7.6;10.3)	8.3 (7.4;9.2)	ns	8.6 (7.5;10.5)	8.3 (7.5;9.3)	ns	9.4 (7.6;10.9)	8.4 (7.5;9.7)	ns	8.9 (7.7;10.7)	8.3 (7.5;9.7)	ns
Z (Ω)	540 (493;611)	550 (469;626)	ns	483 (426;525)	494 (452;520)	ns	470 (426;523)	494 (447;531)	ns	475 (428;523)	487 (432;526)	ns	454 (434;522)	487 (430;526)	ns
R (Ω)	469 (421;515)	492 (438;528)	ns	479 (422;521)	490 (449;517)	ns	466 (422;519)	490 (443;528)	ns	472 (423;519)	482 (428;521)	ns	450 (429;518)	483 (427;521)	ns
Xc (Ω)	63.6 (57.0;69.8)	64.2 (54.3;71.5)	ns	64.0 (57.0;71.8)	60.2 (53.1;67.3)	ns	63.6 (57.0;69.8)	64.2 (53.6;71.5)	ns	63.0 (59.4;68.4)	64.0 (55.2;72.1)	ns	62.4 (59.4;67.8)	64.0 (55.0;72.2)	ns
PhA	7.78 (7.11;8.32)	7.24 (6.83;7.78)	0.009	7.58 (7.05;8.18)	7.03 (6.21;7.57)	0.005	7.66 (7.08;8.23)	7.20 (6.9;7.9)	0.017	7.53 (7.11;8.57)	7.38 (6.91;8.06)	ns	7.56 (7.12;8.24)	7.34 (6.9;8.05)	ns
PA-EE	36.8 (34.9;42.4)	37.3 (35.0;41.4)	ns	37.0 (35.0;41.9)	37.4 (34.8;41.3)	ns	37.3 (35.2;43.1)	36.6 (34.4;41.3)	ns	36.9 (34.4;44.7)	37.0 (35.0;41.4)	ns	40.2 (35.2;44.7)	36.5 (34.5;40.8)	0.043
PA-HRB	2.0 (1.0;3.0)	1.0 (0.0;2.0)	0.001	2.0 (1.0;3.0)	1.0 (0.0;1.0)	0.0005	2.0 (1.0;3.0)	1.0 (0.5;2.0)	ns	2.0 (0.5;3.0)	1.0 (1.0;2.0)	ns	2.0 (1.0;3.0)	1.0 (0.0;2.0)	0.017

MNA: Mini Nutritional Assessment; BMI: body mass index; WHtR: waist-to-height ratio; FM: fat mass; FFM: fat-free mass; RMR: resting metabolic rate; TBW: total body water; ECW/ICW: extracellular water and intracellular water ratio; BCM: body cell mass; SMI: skeletal muscle index; Z: impedance; R: resistance; Xc: reactance; PhA: phase angle; Ω: Ohm; VAS scale: visual analogue scale; PA-EE: physical activity energy expenditure; PA-HRB: physical activity health related behaviours; NH: nursing home group; CD: community- dwelling group; ns: not significant.
